# Harms to “Others” and the Selection Against Disability View

**DOI:** 10.1093/jmp/jhw067

**Published:** 2017-02-09

**Authors:** Nicola Jane Williams

**Affiliations:** Lancaster University, Lancaster, United Kingdom

**Keywords:** *distributive justice*, *person-affecting harm*, *personal autonomy*, *pre-natal testing*, *reproductive technology*

## Abstract

In recent years, the question of whether prospective parents might have a moral obligation to select against disability in their offspring has piqued the attention of many prominent philosophers and bioethicists, and a large literature has emerged surrounding this question. Rather than looking to the most common arguments given in support of a positive response to the abovementioned question, such as those focusing on the harms disability may impose on the child created, duties and role-specific obligations, and impersonal ‘harms’, a less commonly made set of arguments is focused upon which looks to the harms that a decision not to select against disability may impose on others. Three different possible arguments supporting a limited duty of disability avoidance are thus identified and subsequently explored: harms to parents themselves, harms to existing family members, and harms to other existing members of society.

## I. INTRODUCTION

The reproductive realm—historically characterized as overwhelmingly dominated by chance—has steadily become an arena over which individuals have the potential to exercise a significant degree of choice and control. In general terms, shifts in public and individual attitudes as well as the liberalization of abortion laws and the ready availability of contraception have meant that in many nation-states, persons can now choose when and whether to procreate. More specifically, significant increases in human knowledge regarding the mechanisms of inheritance and the subsequent development of carrier, preimplantation, and prenatal testing technologies have also greatly increased the potential for reproductive choice where such choice is desired.

Such technologies have been used for a number of purposes such as sex selection for reasons of “family balancing” or personal preference and to create children who are tissue-matched to existing siblings. But although this is so, the value of their availability—in terms of the creation of additional and meaningful choice—is often thought to be most clearly seen in their use for the purpose of selection against disability and disease. For, while historically, individuals at a greater risk than average of conceiving and birthing children with serious genetic diseases or disabilities, and *who would prefer not to do so*, were faced with only three real options when it came to reproduction: to remain childless, to adopt, or to gamble and reproduce naturally; the availability of genetic testing technologies has meant that the choices available to such persons regarding reproduction have greatly increased. Couples or single reproducers, after all, may now make a considered choice not only among the options historically available to them but also whether or not to utilize donor gametes after carrier testing has revealed a high likelihood of disease or disabling genetic traits in a fetus/embryo, which embryos to implant using in vitro fertilization (IVF) after preimplantation genetic diagnosis (PGD), and whether or not to continue with a pregnancy after prenatal testing has revealed the presence of such traits in a fetus. Indeed, such technologies have also provided prospective parents with the opportunity, should they desire to make use of it, to prepare for the birth of a child who may have complex medical or social needs.

Yet although in many senses the increasing availability of genetic testing technologies should be seen to be autonomy-fostering, it has also prompted many to consider the relative moral obligations of prospective parents toward both their potential offspring and society as a whole and to ask what should and should not be done with the knowledge that may now be acquired. The questions raised by such technologies are many, ranging from the rather generic yet still unresolved question of the moral status of the human embryo/fetus to fears about discrimination against those with “undesirable” characteristics and slippery slopes into a Gattaca-style situation of designer children and a genetic underclass. Unsurprisingly, then, some have questioned whether such selection technologies and techniques should even be available to prospective parents and objected to their use in all or most circumstances for a number of moral reasons.^1^ However, while such contentions are undoubtedly interesting and warrant close examination elsewhere, this paper is concerned only with one form of selection: selection against disability, and whether prospective parents may not only be permitted—it *will* be assumed that they should—but morally required, at least in certain circumstances, to avail themselves of such technologies and take active steps to screen out disability in their offspring.

This question has been raised in the media in response to cases of *selection for* traits widely viewed to be disabling. The most high profile of such cases is that of Sharon Duchesneau and Candace McCullough, a deaf couple who ensured the birth of two deaf children in the early 2000s via the use of a fifth-generation deaf sperm donor (Spriggs, 2002, 283–87). In this case, it was asked whether the couple might have been morally obliged to select a hearing donor in order to increase their chances of conceiving hearing children or to use PGD in order to select a hearing embryo. Indeed, so strong was the public reaction to this case that in 2008 the law governing the uses of assisted reproductive technology in England and Wales was amended to include a clause prohibiting their use for the purpose of selection for disability (Parliament of the United Kingdom, 2008, 10). This question has also been raised in cases where individuals know they are at a *greater than average risk* of conceiving offspring who are likely to be disabled and choose to leave their offspring’s health to chance rather than avail themselves of selection techniques such as gamete donation, PGD, and prenatal diagnosis to lessen that risk. A good example of such a case is that of Bree Walker Lampley, an American television personality with Ectrodactyly who “became the subject of a public discussion about whether it was appropriate to conceive a child who faced a 50-50 chance of inheriting the same condition” (Charo and Rothenburg, 1994, 105), after she was condemned on a nationally broadcast radio show for her decision to reproduce (Parens and Asch, 1999, S10).

Indeed, a preference for selection against disability has also become evident in public attitudes toward those who, despite no knowledge of increased risk, unexpectedly discover genetic abnormalities liable to result in disability in a fetus. Dena Davis, for example, has noted that, despite the fact that 20 years ago on seeing a woman in the supermarket with a child who has Down’s syndrome her “immediate reactions were sympathy and a sense that that woman could be me,” she is now “more likely to wonder why she didn’t get tested” (Davis, 2001, 18). Such an observation on Davis’s part, while not a direct condemnation, does seem to suggest that, for some, selection against disability is no longer viewed as just one among many equally acceptable actions a woman may take upon discovery of fetal abnormality but as the most desirable of such routes. Indeed, less subtle and more striking is the example of the recent media furor surrounding some remarks made by the evolutionary biologist Richard Dawkins on a social media site, where he suggested that to bring a child with Down’s syndrome into the world when one could have had an abortion constitutes an immoral act (Dawkins, 2014).

The question of whether prospective parents might have a moral obligation to select against disability in their offspring has thus, unsurprisingly, piqued the attention of many prominent philosophers and bioethicists, and a large literature has emerged on this topic. Some have couched their discussions in positive terms, asking if there might be a moral obligation to create *the best child possible*. In most cases, however, regardless of whether the question is framed positively or negatively, it is evaluated by consideration of values such as parental autonomy, the welfare of the child created, parental duties and obligations to their children, and considerations of impersonal harm. However, what, at least in part, seems to be the focus of this question, although it is often only alluded to in the literature, are the harms that certain disabilities have the potential to impose on others. In light of this, this paper asks a slightly different question to the usual one in this area. It asks, might prospective parents have a limited moral obligation to select against disability in virtue of the *person-affecting harms* that the creation of disabled persons might impose on those other than the child created?

## II. THE SELECTION AGAINST DISABILITY VIEW AND ITS VARIANTS

Before discussing whether there might be good moral reasons in favor of selecting against genetic traits liable to result in disability in our offspring, it is important to define first what exactly is meant in this paper when it is stated that some particular person is, or will be, disabled. What constitutes disability and whether or not it is intrinsic (a result of individual limitation) or extrinsic (a result of the responses of others and society) to those who experience it is heavily contested, and countless attempts have been made to provide an uncontroversial account. However, for the purposes of this paper, a more general and hybridized definition of disability that fits with current societal and legal conceptions of disability will suffice. Taking as a basis the definitions of disability provided by the World Health Organization^2^ and the 2010 Equality Act,^3^ the term disability here refers to the substantial and long-term negative effects, such as activity limitations and participation restrictions that result from the interaction of individuals with physical or mental impairments and other health conditions with their environment, and a genetic trait will be referred to as disabling when it is liable to result in the existence of a person who fulfills such criteria.

The Selection Against Disability View should be described as encompassing a family of views which pick out one or some number of negative characteristic/s that is/are perceived to attach to disability (e.g., limited opportunity or suffering) and hold, in virtue of this association, that, ceteris paribus, it is morally preferable to choose to bring to birth a child absent this/these characteristic/s. It is *not* a monistic view, and views falling under its umbrella need only exhibit a weak commitment to the belief that knowledge of the fact that a fetus/embryo is in possession of genetic or physical traits liable to result in disability constitutes good reason, although that reason may be defeasible, against a decision to bring it to birth. Thus, subscription to such a view does not require one to hold that it will always be morally wrong to choose to bring to birth a child with a disability, the strongest and least plausible version of the Selection Against Disability view, nor, indeed, even for one to subscribe to the view that it will *normally* be morally wrong to fail to do what one can to ensure that one brings to a birth a nondisabled child, although such views are, of course, included within its umbrella.

In this rather general form, we can note that the Selection Against Disability view as it is characterized here is just vague enough to offer *very* little guidance as to when selection should be deemed morally required and when a failure to select against disability would be deemed morally acceptable, in virtue of competing normative claims.^4^ This is so for good reason. For, while the abovementioned definition and explanation captures the general view held by those who subscribe to this view, it is the case that, depending on the variant to which one subscribes and the circumstances in which we find ourselves, incredibly different answers will be given to the question of whether it is morally acceptable for person (p) to fail to select against disability or to deliberately select for disabling trait (x) in some particular scenario (s). This vagueness takes into account that different variants provide different reasons as to *why* it should be considered normatively preferable to select against (x) in one’s offspring. These reasons, unsurprisingly, pick out different *elements* as providing the wrong-making quality of failing to avoid disability in one’s offspring and thus hold different amounts of weight when pitted against competing moral claims, such as the emotional burdens, physical risks, and financial costs a prospective parent may have to bear in order to fulfill its prescriptions.

Arguments that may form the basis of a version of this view can generally be seen as falling into four distinct categories focusing on different justifications. The major positions associated with such justifications shall now be outlined before moving on to focus on the fourth in order to provide a basis for our discussions:

1. Harming and Wronging the Child
2. Violations of Parental Duties and Virtues
3. Impersonal Harms
4. Harms to Others

The first class of argument suggests that a duty of disability avoidance can be grounded in the interests and/or the rights of the children that will result from our procreative decisions. Arguments falling into this category thus focus on person-affecting reasons, picking out the harms or limits to opportunity associated with certain disabilities as providing good moral reason to select against traits liable to result in such harms and limits to opportunity. Perhaps the most straightforward argument of this type would be that as disability is, by definition, a harmful or disadvantageous state for an individual to be in, it would be *better for* some/all disabled fetuses/embryos, should they not be brought into existence. This, however, requires one to commit to the claim that the lives of disabled individuals are often dominated by suffering and has very few, if any, proponents. For, to say a life is not worth living places a large burden of proof on those who make such a claim. As such, it is generally assumed that arguments focusing on the harms that disability may impose on our children may legitimately be made, if at all, only in cases of extremely serious and/or painful disabilities.^5^

Others attempt to lessen the threshold level of disability, suggesting a version of the selection against disability view may be grounded in the rights of those who may be brought to birth as a result of selection decisions. The most famous of such approaches is Davis’s application of Feinberg’s Open Future Argument to the prenatal context. She suggests that a decision to implant an embryo that may be subject to a genetic disease over a healthy embryo violates the prospective child’s “rights in trust” (Davis, 1997, 7–15). Such rights are held to be a corollary of the autonomy rights that adults possess in liberal societies and require that parents not close off certain important choices their children may wish to make when they are adults while they are still minors (Feinberg, 1992a, 80).

Arguments falling into the second category look to the kinds of role-specific duties that parents *owe* to their children (Hare, 2007; Wolf, 2009) and the kinds of virtues that a good parent should possess (McDougall, 2005, 602–603). They focus, like the first, on the harms and limits to opportunity that may result from the possession of disabling genetic traits, arguing that a good parent should not want to create a child who is likely to be disadvantaged in such a way. This is the case with Steinbock and McClamrock’s “principle of parental responsibility” which requires persons to “refrain from having children unless certain minimal conditions can be satisfied . . .[as] loving, concerned parents—will want their children to have lives *well* worth living” (Steinbock and McClamrock, 1994, 17).

Arguments falling into the third category appeal to nonperson-affecting concerns, such as the effects of a failure to select against disability on the state of the world by arguing that the creation of persons with disabilities may in some or other way make the world a poorer place. Parfit, Glover, and Harris, for example, have all famously argued that states of affairs need not be good or bad *for* anyone in order to be morally blameworthy but may instead be good or bad *tout court*, and they have applied this reasoning to the question of whether there might be a moral obligation to select against disability. They argue that those who fail to avoid disability in their offspring act wrongly because they deliberately or negligently choose to create a state of affairs that is strictly worse (in terms of some particular value such as happiness/preference satisfaction) than the state of affairs that could have been created, had they chosen differently (Parfit, 1984; Glover, 2001; Harris, 2001). This kind of argument can be seen in Savulescu’s Principle of Procreative Beneficence. It states that prospective parents have a duty to “select the child of the possible children they could have, who is expected to have the best life, or at least as good a life as the others, based on the relevant available information” (Savulescu, 2001, 415), a duty which, although not limited to selection against disability, would require, in the vast majority of circumstances, such selection. It can also be seen in the eugenics policies of the late nineteenth and twentieth centuries that attempted to build a “better” society via the implementation of selective breeding programs and the forced sterilization of those deemed genetically undesirable.

Finally, the fourth class of reason, like the first, consists of arguments suggesting that a duty of disability avoidance may be grounded in the person-affecting harms liable to result from a failure to select against disability. Unlike the first class, however, variants falling into this category should be seen to focus outwardly on the harms—financial, emotional, relational, and otherwise—that a decision to select for/not to select against disability may impose on persons already in existence, that is, on individuals other than the fetus itself.^6^

In recent years, much attention has been given to the first three of the abovementioned classes of reason. Both those who champion and criticize a version of the selection against disability view have written extensively on such matters, as, indeed, have the many authors who have written papers offering both in-depth and surface surveys of different incarnations of this debate.^7^ Yet, despite this, arguments falling into the fourth category have been largely neglected^8^ in the literature. Why this has been the case, however, is mysterious, and we argue that this is so for two reasons.

First, because there is a general tendency within society and philosophy to question the moral acceptability of acts including but not limited to choices to engage in extreme and dangerous sports, go on exotic holidays, smoke, or eat unhealthily. From the perspective of analysis of the externalities such choices produce, it seems bizarre that acts of procreation should be immune from criticism on the same grounds. Second, it should also be noted that it is now generally held that arguments focusing on harms to the child fail to provide a defensible moral foundation for the selection against disability view in all but the most severe cases of disability, in virtue of the conclusions of the nonidentity problem. Thus, most theorists have only two welfarist options when it comes to possibilities for a moral grounding for such a subscription: appeals to impersonal harms and harms to others. That impersonal harms have received more attention than those affecting actual persons constitutes the second mysterious reason. For, even if impersonal harms are possible and relevant when determining the moral status of our actions, acts causing harm to actual persons are generally held to be of more moral gravity than those causing impersonal harms. As such, if a failure to select against disability can be shown to cause unjustifiable harms to actual persons, it may be the case that such harms can provide a stronger moral basis for a duty of disability avoidance.

With this in mind, and taking as a basis a comparative account of harm as setbacks to interests—according to which it is held that some particular entity (p) is harmed by some particular action (x) when it is the case that (x) has adversely affected the interests of (p), or counterfactually, when it is the case that the interests of (p) are, all things considered, in a worse condition than they would have been, had (x) not occurred (Feinberg, 1992b, 7)—I gradually widen the area of concern from which I analyze the real or perceived harms that a failure to select against disability have the potential to impose on others. Where such harms are found, I then ask whether they might be deemed wrongful, providing a defensible moral foundation for a version of the selection against disability view. Thus, I begin by looking to the harms that prospective parents might be said to impose on themselves by a failure to select against disability. The focus is then widened slightly to include family members whose interests might be negatively affected by such a decision. Finally, after this is done, I widen the focus to its furthest reach, looking to the idea that a failure to select against disability may cause unjustifiable harm to our fellow citizens in virtue of the moral claims that such decisions impose on them regarding the proper and just division of social resources.

## III. PERSONAL HARMS: AUTONOMY AND THE COSTS OF PROCREATION

Whatever the reasons we have for reproducing, very few of us do so because we believe that in procreating we will benefit our future children. Nor, indeed, do any but the most collective-minded of us decide to reproduce for the reason that we believe our child’s existence will provide some benefit to humanity. Instead, our decisions to do so tend to be based in considerations of self-interest, individual perceptions of obligation, or a-rational in nature, undertaken with no specific purpose in mind but unthinkingly and perhaps out of a sense of inevitability.

We may believe, for example, that in undertaking the project of parenthood we will be better off; that we might find love, fulfillment, companionship, or a sense of purpose; and that a child might provide the antidote to an ailing marriage or provide us with security and support in our senescence. We may instead, or in addition, view that there is an obligation on our part to continue the family line or name, to provide our partners or parents with children, believe that parenthood is socially or culturally required, or perceive a religious obligation to “be fruitful and multiply.” We may, too, find that reproduction is not really a “choice” at all, because we find ourselves pregnant and/or in possession of beliefs that tie our hands or because we have been conditioned to think that to have a child is, in some sense, inevitable, not required as such, but a natural part of human life that we tend not to fail to avoid except on significant motivation.

For the self-interested among us who procreate not merely out of a sense of obligation or arationally, it can be noted that decisions to do so tend to be based on a belief that, in accordance with the information we have available, we will be better off should we conceive/give birth to/raise a child, or, at the very least, will not be caused harm by our decision on balance. Thus, we accept (implicitly or explicitly) the costs of parenthood, such as the time, money, and effort that it takes to rear a child, in anticipation that the benefits we seek will outweigh such costs. Yet, despite this, in certain scenarios a decision to bring some particular child to birth may leave us worse-off than we would have been, had we chosen differently, and to make such a decision, when in full knowledge of this fact, will be one that cannot be considered rational if we are seeking to act self-interestedly. As such, it may be possible to claim that in cases where a reproductive choice (including but not limited to a decision not to select against disability) will leave prospective parents worse off than they would have been, had they made a different choice, such individuals might well wrong themselves.

Whether or not such a claim can be made, however, is determined, at least in main, by our responses to two questions. The first of these regards what we view to constitute the *components* of an autonomous decision. The second concerns our *ability* to both predict and rank different possible states of affairs relating to the choice of whether or not to select against disability such that we may show that the life of some individual is liable to be *worse*, as opposed to merely different, as a result of this choice.

Regarding the first question, if it is held along broadly Millian lines that it is the possession of certain capacities (such as reason, reflection, and representational abilities), as opposed to their exercise, that constitutes autonomous choice, then concluding that a given decision is irrational does not provide a reason to claim that those who make irrational choices either wrong themselves or may be wronged by others who fail to stop them from acting irrationally. Provided an individual possesses such capacities, is not being coerced by others, and is in possession of the information required to make an autonomous decision, he may just as easily choose to cause himself harm as he may to furnish himself with benefits. This is so, as if the moral agent in such cases were the same as the moral subject, the idea that he might act wrongly by harming himself constitutes a contradiction. For, the moral subject, in his position as moral agent with the capacity to release others of their moral obligations to him, has the capacity too to release himself from his own.

On other accounts, however, such as in the position taken by Kant regarding the possibility of duties to self, this contradiction does not occur. On this account, persons are dualized entities, the *homo noumenon*, a perfectly rational legislator, and the *homo phaenomenon*, a finite and imperfect being who, by possessing the capacity of free will, may both obey the law of the former and fail to obey it. Such entities thus stand together, creating a state of dialectical opposition between the demands of duty and the pull of inclination (Kant, 1996, 6:418–420). On such an approach while one, as the latter entity, can make an autonomous choice and consent to doing the irrational, one cannot as the former, in virtue of the fact that the perfectly rational legislator *cannot,* consent to the irrational. By acting only as the latter entity and ignoring the prescriptions of the prior, one can wrong oneself, just as one can wrong others, by failing to act in accordance with duty. As such, those who subscribe to this view may claim that in situations where one has a choice between a rational and an irrational procreative decision, to choose to act irrationally will constitute a moral wrong.

One need not subscribe, however, to the Kantian account to locate the wrong of an irrational procreative decision in the harms that a procreative decision maker may impose on himself. On other “thick” accounts of autonomy, for example, we may claim that the individual who “chooses” to act irrationally actually makes no choice at all because an autonomous choice *is* a rational one. Irrational choices are necessarily inauthentic in the sense that the decision maker either lacks the information sufficient to make the “right” (rational) decision or his capacity to understand that information has been compromised. Thus, although an agent will not wrong himself, others might well be said to wrong him by failing to intervene and stop him from harming himself in such scenarios.

Should we adopt such an approach, however, this may still not justify the *outward* imposition on those who would make such decisions of a moral duty to act in a rational manner. For, while paternalistic attempts to save individuals from themselves may have their place in the writings of Rousseau (1997) and Plato (1993), they are at odds with the insistence common to most, *if not all*, mainstream ethical theories, including that of Kant himself, that we should aim to preserve and enlarge the arena of his life over which the individual can be said to be sovereign. In accordance with this commitment, it is held that ceteris paribus individuals should be given the freedom—both morally and politically—to make their own decisions regarding *how* to live their lives, regardless of how foolish others may view such decisions to be (Mill, 1989; Kant, 2005). Indeed, oppressive moral, social, and political environments have proven themselves time and again throughout history not to be particularly conducive to individual well-being. Individuals are, after all, normally in a better place to judge the extent to which their decisions might cause them harms and benefits than those who are ignorant of the individuals’ own mental states (such as their beliefs, desires, and emotions). Indeed, even where this is not so, it can be noted that individuals tend to give special weight to decisions they make themselves: gaining more pleasure and pride from their own successes and accepting more readily the harms they impose on themselves than those imposed from outside.

However, although it can be claimed on certain accounts of autonomy that those who harm themselves act wrongly or may be wronged by our allowing them to do so, it is yet to be demonstrated that those who choose not to select against disability in their offspring are actually liable to be made worse-off than they would have been, had they chosen differently. Whether or not this will be the case, of course, can only be predicted, due to the fact that certain knowledge of the future is impossible. Indeed, neither is a positive judgement of wrong in one specific case always likely to lead to a positive judgement in another. For, our ability to make such a judgement is greatly dependent on a large number of factors that may interact with one another in a number of ways, such as the nature and severity of the disability in question, the identity of the prospective parents, and the harms they may suffer as a result of selection as well as the structure of the society in which the child will be raised, and so on.

In favor of this judgement, it can be noted that it has been well documented that raising a child with a disability often proves *more costly for parents* than raising a child who is not disabled. Economically speaking, on top of the normal costs associated with raising a child, depending on the nature and severity of the disability, parents may need to make significant adjustments to their homes. They may need to purchase special equipment, adapted and/or specialized toys, food, and medicines. One or both parents may need to stay at home to care for the child, lowering their earning potential and resulting in less financial resources to satisfy their own interests (Dobson and Middleton, 1998). Emotionally and socially too, the stress of dealing with these financial costs may take a toll on their relationships; without adequate support from others, they may feel isolated, and the pain of seeing their child struggle with the mastering of tasks that come easily to other children or watching them suffer from the painful effects of certain disabilities may also prove detrimental to their welfare.

Yet, despite the fact that empirical evidence shows that there are often extra costs associated with the raising of children with certain disabilities, it is still to be demonstrated that such costs are not likely to be outweighed by compensating benefits. In the case of persons who wish to select for disability in their offspring, for example, this seems, for obvious reasons, to be untrue. Such individuals, in making a concerted effort to create a child with a particular set of genetic characteristics, tend to express a belief that raising such a child will, *for them,* be just as, if not more, fulfilling, worthwhile, or enjoyable than raising a child without the particular characteristics they seek.

Even in cases of severe and unchosen disabilities, that the birth of a disabled child is likely to prove harmful on balance or, in a stronger sense, produce a less favorable ratio of benefit to burden for his parents is highly questionable. It may, for example, be claimed that in comparing and ranking as better or worse the possible states of affairs resulting from a decision whether or not to select against disability we are erroneously assuming that such states of affairs are commensurable when, in fact, they are not. It may be claimed, for example, that, just as the concept of the good life is too rich and complex to allow that one life may be determined better than another by reference only to the benefits and burdens it contained, it is impossible, too, to rank the states of affairs resulting from a selection decision on this basis. Indeed, a review undertaken of studies regarding the impact on family life of parenting a child with a severe disability notes that despite such costs the lives of parents of children with disabilities tend to resemble the lives of parents generally (Ferguson, Gartner, and Lipsky, 2000, 73). In addition, another study comparing child-related and parenting stress in parents of children with and without disabilities notes that parents of disabled children “exhibit variability comparable to the general population with respect to important outcomes such as parental stress . . . family functioning . . . and marital satisfaction” (Krauss, 1993, 393–404). In other words, while parenting a child with a disability might pose certain challenges—especially in terms of finances—because society tends not to provide the conditions conducive to the trouble-free rearing of severely disabled children (Kittay, 2000, 167), the act of parenting a child with a disability seems to be no more or less likely to be fulfilling, unfulfilling, stressful, enjoyable, damaging to one’s relationships, or difficult for parents on balance than parenting a nondisabled child.

Finally, depending on the timing and method of selection, the act of selection against disability has the potential, just as might the raising of a child with a disability, to impose great harms on prospective parents. Thus, a decision to choose not to select against disability even in the face of great burdens may well, for some, prove to be the least harmful option available to them. When regarding abortion after the discovery of fetal abnormality, for example, for those who subscribe to a prolife view, feelings of guilt and shame might be overwhelmingly strong and thus override any benefit that may be produced by procuring an abortion. Indeed, even where this is not the case, it should be noted too that gestation involves a great deal of intimacy between the fetus and the mother. For, as has been noted by Anstey, “Gestated entities are strongly incorporated into the mother’s body and especially subject to bonding relationships” (Anstey, 2008, 237). As such, the decision to abort a fetus after a diagnosis of a genetic or developmental abnormality may be one that would cause a great deal of lasting psychological pain that, depending on its intensity, could outweigh the benefits the performance of an abortion would produce.

## IV. SELECTION AGAINST DISABILITY AND THE JUST DISTRIBUTION OF FAMILIAL RESOURCES

Attempts to ground a version of the selection against disability view in the harms that raising a child with a disability may impose on parents themselves seem, for the reasons outlined earlier, to be unlikely to succeed. However, although parents may be said in the vast majority of cases to accept the harms and benefits they impose on themselves by their procreative choices, reproductive decisions, like all others, do not take place in a vacuum. They affect, for better or worse, not just ourselves and the objects of our procreative efforts, but virtually all members of society, producing harms and benefits that, regardless of whether we intend them or not, *will* be relevant for determining the moral status of any particular procreative act.

The idea that others might be harmed to the extent that a decision not to select against disability might actually constitute a wrong seems most likely to hold in situations where our existing dependents may be negatively affected by our choices. For, we often view that we owe special moral obligations to family members that extend beyond our obligations to strangers, and even where this is not the case can note, too, that family members, in virtue of their close proximity to us and our choices, are more likely than others to be significantly affected by them. A case might thus be made for a moral duty to select against disability in situations where a decision not to do so would be made by those with existing children or who have taken on responsibility for ensuring the welfare of dependent adults. For, while the addition of a new member to a family will, in most cases, affect the interests of existing members, as both familial resources and the time and attention spent satisfying and nurturing the interests of existing dependents will have to be spread more thinly, when the new member of a family suffers from a disability, such issues may be compounded. Disability costs, and parents bear a significant proportion of these costs even in societies such as our own, where certain of the costs associated with child rearing and disability are socialized. As such, these costs, if prospective parents have any, will affect their existing dependents. The needs of an infant with a disability may be far greater than the needs of one without and may sometimes not reduce as the infant becomes a child and that child becomes an adult. This means that in such circumstances the needs of one’s other children will often be addressed only after the needs of the child who is disabled.

This is not, in itself, necessarily morally problematic. For, provided we hold that familial resources *should properly* be diverted to those with the most need for them, it will be the case that to take one’s eldest child to ballet lessons, to save money for her university years, or to help her with her homework, although good things to do, must come second to ensuring that the safety and basic needs of one’s other dependents are met. This view is illustrated well by Nagel in the following example:

Suppose I have two children, one of which is normal and quite happy and the other of which suffers from a painful handicap. . . . I am about to change jobs. Suppose I must decide between moving to an expensive city where the second child can receive special medical treatment and schooling, but where the family’s standards of living will be lower and the neighbourhood will be unpleasant and dangerous for the first child—or else moving to a pleasant semi-rural suburb where the first child, who has a special interest in sports and nature can have a free and agreeable life. (Nagel, 1978, 22)

Nagel argues that in this case even if the benefits of moving to the semirural suburb for the first child will be far greater than the benefits that the second child will receive by a choice to move to the city, it will be the case that as the needs of the second child are more urgent he should still choose to move to the city. Provided the welfare of the first child does not fall below some threshold level such that he would be made worse-off than the second child by the move, or the benefits accrued by the second child are so minimal that they are virtually nonexistent, a decision to place the interests of the second child over those of the first child will be morally required (Nagel, 1978, 23–24).

Yet, some such as Roberts have noted that whether or not we view that parents should properly distribute benefits and burdens unequally between their offspring when one is disabled and the other is not is irrelevant. For, she notes in reference to a case similar to Nagel’s where an existing sibling is expected to make sacrifices for the welfare of one who is disabled, differing only in the sense that the parents *make a conscious decision* to bring to birth a child with severely disabling genetic characteristics:

That the parents, having chosen to produce the impaired child, then make the *further* choice to distribute wellbeing appropriately between that child and others—and hence, not to unjustifiably harm those others by that further choice—does not imply that the harm imposed by the *original* choice can itself be justified. There are two distinct choices—and two distinct distributive effects. (Roberts, 2009, 22)

Put more simply, the claim Roberts makes is that in cases such as that mentioned earlier, the choice to bring a child who will be severely disabled into the world is that which is under scrutiny, not how to distribute resources or welfare within a family once that decision has been made. Roberts thus argues that when we assess the rightness or wrongness of a particular procreative choice, we must compare “(1) the effects of that choice on each person, against (2) the effects of *each alternative choice*, including those that *exclude* bringing [the impaired child] into existence” (Roberts, 2009, 29). This leads her to suggest that in cases when prospective parents choose to bring into existence a child with a *serious* disability that will impact on their ability to create well-being, opportunity, and so on, for an existing dependent, such a decision will constitute a harm for him. For, regardless of whether the parents can be said to distribute well-being and other resources appropriately between him and his sibling once born, it is still the case that he can claim that he would have been better off, had they chosen differently.

Putting aside the epistemic questions that surround cases such as the one Roberts envisages—concerning when and whether it will be possible in reality to predict with accuracy that a particular reproductive choice is likely to result in harm to an existing child—whether or not harms to existing dependents where they can be predicted will constitute a wrong depends greatly on what moral obligations parents have toward their offspring. For although “parents who decide to bring a child into the world have special duties to that child because, in deciding to procreate, they take upon themselves responsibility for this child’s well-being and development” (Blustein, 1992, 228), there are many competing accounts of the kinds and extent of obligations they consent to assume.

Do those who take on the role of parent, for example, have a moral duty to always do the best for their children, to act in their best interests regardless of the sacrifices they themselves must make? If such is the case, it would seem that in cases where prospective parents have existing dependents, a decision to bring to birth a child with a disability is one that should be made very carefully because it has the potential, in many circumstances, to constitute a moral wrong. Yet, on such a strong account of parental obligation, it would seem that parents are constantly at risk of unjustifiably harming their children by their choices, reproductive or otherwise. Thus, in the case of reproductive choices, just as we might ground a moral obligation to select against disability in the interests of existing children, so too might we ground an obligation to have or not to have additional children, disabled or not, in such interests if it can be shown, for example, that only children will tend to be worse or better off than children with siblings. Indeed, just as the sibling of a child with a disability *may* be more likely to feel ignored or neglected by his parents than siblings of children without disability, the same could be said of siblings of gifted children. For, studies have revealed that relations between nongifted and gifted children are less intimate than those between nongifted siblings and are often characterized by jealousy and resentment of their gifted sibling’s “arrogance” and intellectual abilities (Lapidot-Berman and Oshrat, 2009, 36). In cases not related to reproduction, it can be noted that such a strong account of parental obligation might require loving but poor parents to hand their children over to equally loving and wealthy adopters if such persons are in a better position to care for them and that the parents of incredibly intellectually gifted children might unjustifiably harm them by failing to mortgage their homes in order to provide an exclusive and incredibly expensive education.

Indeed, any decision that parents make, taken with their own, or the interests of others, as opposed to their child’s, in mind would seem to be vulnerable to moral criticism because the parents would be, in virtue of their role, morally responsible for any and all decisions with less than optimal results for their child. Similar problems seem to plague Feinberg’s account of parental obligation, which requires that parents send their children out into the world “with as many open opportunities as possible, thus maximising [their] chances for self-fulfilment” (Feinberg, 1992a, 84). With this in mind, it is suggested that a defensible account of parental obligation must take both a weaker and a more complicated form than the requirement to always act in the best interests of one’s children or to furnish them with maximally open futures. Such an account *should* take into consideration the interests of the parents themselves but should also, and importantly, prove compatible with the moral claims of other members of the society.

The extent of parental obligation, however, still differs greatly on different accounts. Some, for example, suggest only that parents satisfy their children’s basic needs or that their children reach some minimum threshold level of well-being. Others hold that parents must *do what they can* to ensure that their children have good lives, and others still make less concrete claims regarding a parental obligation to “love” or to exhibit a kind of “natural affection” (Hume, 1978, 478) that requires personal sacrifice and must be sustained even in the face of difficulties that might destroy most relationships.

On the most minimal conception of parental obligation, we can note that parents will be required merely to satisfy their children’s basic needs for food, shelter, education, clothing, and comfort until their child is able to satisfy such needs himself and that any other benefits they may choose to bestow on their child should be seen as supererogatory. On this account, unless a parental decision, in some way or other, will cause parents to be unable to meet these basic needs, their choices would be deemed irrelevant, morally speaking, in respect to their role. As long as a decision to have another child, disabled or not, will not impact negatively on parental ability and willingness to attend to such needs so that one’s existing child may claim “I would have been better off, had you not acted in the way you did,” the decision will not constitute a valid moral complaint and will therefore not provide a moral reason to select against disability.

Indeed, even on the more substantial accounts noted above where parental obligations extend to loving and forwarding certain of their children’s less basic interests, we should note that the comparative harms prospective parents may impose on them by a decision not to select against disability may well be quite high. Requiring parents to do their best to ensure their child has a good life is, after all, far less demanding than a requirement that we ensure he have the best life available to him, and so too is the demand that parents exhibit a kind of sacrificial love when making decisions that impact on their children.

Indeed, it should be noted, too, that cases such as those Roberts mentions—where it can be predicted quite accurately prior to birth that a decision to bring to birth a child with a disability will impact negatively on the interests of an existing dependent—are likely to be far fewer in number than might be assumed. For, in cases of less severe disabilities and conditions such as Down’s syndrome, which can come in both mild and severe forms, it may be virtually impossible to predict with accuracy the extent to which existing family members will be negatively affected by a choice not to select against disability and that in many cases, with careful planning, it will be possible for prospective parents to meet the needs of both disabled and nondisabled offspring adequately. Further, just as the siblings and other family members are liable to bear the brunt of the negative effects of disability in virtue of their close proximity, so too are they best placed to receive the benefits that may come from a close relationship with a person with a disability such as growth into a kind, mature, tolerant, and considerate member of society who is able to see the value in a wide range of different modes of life (Powell and Ogle, 1985). As such, while it can be argued that parents should take into account the sacrifices their existing dependents may have to make in order to satisfy the needs of an additional family member with a disability, such sacrifices are highly unlikely—even in cases of the most serious disabilities and where selection is unlikely to prove harmful to prospective parents, such as where prospective parents are already undergoing IVF and are, however implausibly, offered PGD for free—to prove decisive.

## V. SOCIETAL RESOURCES AND THE DEMANDS OF JUSTICE

In the previous section, the kinds of harms existing dependents might face as a result of a parental decision not to select against disability were explored. It was noted that, unless we are to subscribe to a remarkably strong account of parental obligation, the kinds of harms to interests that siblings may face as a result of a decision not to select against disability will often be justifiable. For, although parents undoubtedly have obligations to furnish their children with certain goods, the goods that existing children are liable to be denied by such decisions will tend not to be of a kind we generally deem parents morally required to provide. Despite this, however, there is another, more widely applicable, sense in which a decision not to select against disability in one’s offspring might be said to impose unjustifiable harms on others *in certain situations*. For, dependent on the social structures of the society in which we live and the extent to which they can be said to embody socialist/egalitarian as opposed to libertarian ideals, as well as the nature of the particular disability with which we are concerned, a parental choice not to select against disability has the potential to impose (rightly or wrongly) substantial costs on existing members of the society.

### Distributive Justice and Reproductive Choices

In societies with advanced socialized medical and welfare systems, the decision to procreate and rear *any* child is one that is expensive for both parents and other members the of society. Indeed, it only increases in expense, the closer our society comes to embodying egalitarian ideals and the greater the inequities that may be faced by our children. This is so as “virtually everything that goes into the production of us, following conception is something supplied by our parents or by people elected or employed for that purpose. They . . . supply us with our pre-natal environment, our medical care, our schooling . . . and all the rest of it” (Steiner, 2002, 186). Yet, although it is generally thought that the provision of public resources to assist parents in child-rearing and to level-out or compensate for the inequities their children may face is morally required, it is in fact far from clear that those who make a decision to reproduce have an enforceable right to demand that others share in the costs associated with their choice. For, just as in the case of procreative decisions and the just division of familial resources, whether we view that a certain basket of social resources must be provided to a child once born actually has little bearing on the question of whether and when prospective parents are morally justified in imposing this burden of support on other members of society.

While historically theorists of distributive justice have tended to say very little about who should bear the costs of child-rearing, only that such costs must be met by someone, the idea that parents may act wrongly by reproducing when they are unable or unwilling to provide their children with the basket of resources required to satisfy their claims to justice has in more recent years^9^ been explored in some depth by a number of prominent scholars.^10^ Some have provided compelling arguments that parents, *in a truly just society*, should be held responsible for meeting the costs of any claims to justice that their children might have as a result of their voluntary actions due to the fact of their voluntariness. Rakowski, for example, notes provocatively:

If children were purely accidents of nature, entering the world independent of anyone’s choices, one could understand why everyone alive would share a duty to care for them. But children are never accidents in this sense. . . . Because specific people are responsible for their existence and needs, parents alone should bear the cost of compensating their children for any cost they suffer genetically or otherwise. The community may serve as a backstop should parents default on their obligation, but it should not be the principal payor. (Rakowski, 2002, 1365–1366)

In most cases, however, a decision to provide societal support to parents in the upbringing of their children and in meeting the claims to justice of such children while they are children and once they reach majority *can be* justified by appeals to self-interest and, where this is not the case, by appeals to the nonideal conditions under which procreation tends to take place.

In terms of self-interest, for example, it can be noted that although individuals tend not to ask for permission to reproduce, this will not often be morally problematic because reproduction generally produces a positive as opposed to a negative externality. Children do not remain children forever and constitute “the future workforce and taxpayers whose economic contributions everyone, nonparents included, will depend on when they reach old age” (George, 1987, 31). Existing citizens have an interest in investing in the production of healthy, productive, and well-educated citizens because in doing so, they will likely increase the stock of resources available for distribution to themselves. Indeed, because most of us accept the benefits created by the production and rearing of children, it may be said that there is a moral imperative to share in the costs involved in their production. This argument has its basis in the principle of fairness/fair play which requires that “if some people engage in a cost-incurring, benefits-producing cooperative scheme[,] it is unfair to free ride on them, and thus that those who accept the benefits resulting from procreative decisions, have an obligation too to do their fair share in maintaining, or bearing the costs of maintaining, the scheme” (Olsaretti, 2013, 238). There are good pragmatic reasons to adhere to the prescriptions of the principle of fairness, too. For, although reproduction produces seemingly nonexcludable goods, and thus it could be argued that it is in the interests of existing members of society to free ride on reproductive and parental labor, to do so may jeopardize the production of such goods, creating a version of the tragedy of the commons or threatening their privatization.

Indeed, where self-interested reasons do not apply, there exist other reasons to provide societal resources to support those who desire to procreate and who would be unable to meet all of the costs associated with their decision without such assistance. First, we can note that notwithstanding the fact that reproduction can hardly be classified as a basic need—we can survive without children but not food, water, and shelter and are able to “form, to revise and to rationally pursue . . . what we regard for us as a worthwhile human life” (Rawls, 1996, 302) absent the former but not the latter—an interest in bearing and rearing children is not exactly trivial, either. Even if we deny the credibility of accounts of a biological “need” to reproduce, strong social and cultural pronatal messages most definitely contribute to a psychological need and thus to the distress, depression, and feelings of bereavement often experienced by those unable to do so (Lechner, Bolman, and van Dalen, 2007), whether the reasons for this inability are somatic or social in nature. Thus, if as a society we are able to accommodate this need, preventing the suffering of those who wish to reproduce but would be unable to do so without our help, and if such accommodations do not prove prohibitively expensive or deny the satisfaction of the more pressing interests of others, there is good moral reason to do so. Second, the ability of individuals to bear the costs of producing and raising children is often determined in great part by factors over which they have little control. Luck is a pervasive component of human existence and, as such, dependent on the theory of justice to which we subscribe so that those who have done well in the natural and social lotteries may well be morally required to subsidize the ambitions, both procreative and otherwise, of those whose starting positions in society were less fortuitous. This would mean, for example, that the unlucky^11^ should be afforded the same opportunities to procreate as the lucky^12^ where their inability to satisfy the claims to justice of their children are, to a significant extent, the result of poor luck as opposed to considered choices.

Despite this, it does not necessarily follow that such reasons will apply in all cases of reproduction. Pragmatically, for example, it can be noted that there seem to be few good reasons for existing members of the society to contribute to the costs of the production and rearing of children who are highly likely to “have initial life prospects that are sufficiently low (for example, below average) that others will suffer either increased [justice] demands (to help the offspring) or reduced . . . entitlements (because the offspring displace them)” (Vallentyne, 2002, 205). For, such increased demands are likely to lead to “diminished per capita resource availability in the short term but also greater depletion of non-renewable resources in the long-run” (Casal and Williams, 2004, 100) and/or other negative effects when such effects are not outweighed by the production of other benefits or justified by the claims to justice of reproducers.

In many cases, of course, we are unable to determine whether a decision to reproduce is likely to diminish or forward the welfare and resource holdings of others. As such, it might be argued that there are good pragmatic reasons to pool risk and bear the costs collectively in order to preserve a scheme that is, on balance, to our benefit. In other cases, informed predictions can be made based on the information available to us. A decision to have a child (or many children) in a time of famine or when overpopulation threatens and resources are already stretched beyond reasonable limits, for example, is always likely to be one that produces a negative externality. Thus, it can be argued that in such cases there might be a duty not to knowingly disadvantage others by our procreative decisions.

Since the advent of genetic testing technologies, it seems that the same reasoning may well apply in certain cases where reproductive choices are liable to result in the birth of a child who will require costly accommodations or expensive medical treatments in order to fully participate in the society or who is unlikely to be able to participate at all. In many cases of reproduction, the birth of a child with a disability should be seen as a result of poor brute luck, such as when a woman at average or low risk of producing a child with a disability discovers an unexpected genetic abnormality in a fetus, where she unknowingly comes into contact with a teratogenic substance during pregnancy, or where complications during pregnancy and birth result in the birth of a child with a disability. Yet, in others—where a couple or single reproducer selects for disability in their offspring (such as in the case of Duchesneau and McCullough), fails to take reasonable precautions during pregnancy to avoid exposure to situations and substances that may cause disability in their offspring, or makes a conscious decision not to avail themselves of services and technologies that will reduce the likelihood of producing children with disabilities when to do so would not impose substantial or unreasonable costs on them—there does seem to be an important sense in which the disability and its costs for others are chosen. A sense that, although not lessening the claim to compensation on the part of the child created—he is not responsible for his coming into existence and is just as entitled to a certain and already agreed-on level of welfare, opportunity, and resources as any other member of a given society—may well, at least on desert-sensitive accounts of the demands of justice, shift some of the responsibility for bearing such costs onto those responsible for his existence. Thus, in cases where such costs *cannot* or *will not* be borne by reproducers^13^, those members of society left to foot the bill may well complain that in these cases parents may be charged with unjustly “exploiting public resources that were not designed for the purpose of accommodating unique procreative preferences” (Fahmy, 2011, 6), and in cases where such resources are finite seem to display a complete lack of concern for other members of the society who, had a different decision been made, would be entitled to their use.

### Do Persons With Disabilities Actually “Cost More”? Does This Matter?

Whether or not the abovementioned argument will succeed in providing prospective parents with good moral reason to select against disability in their offspring in situations where they have a choice depends, however, on our providing a positive response to two questions.

The first of these is empirical in nature: is a choice not to select against disability or deliberately to select for disability in this particular case likely to produce an all-things-considered burden of support on existing members of society that will not be justified by appeals to the claims to justice of the parents themselves? This question is ideally one for economists to answer as opposed to philosophers. However, while such is the case, the claims to justice of persons with disabilities do seem *generally* to cost more to meet than the claims of those without disabilities.

It can be noted, for example, that while surveys regarding the financial costs of disability for families and society are rarely undertaken, in the United Kingdom, parents of children classed as “seriously disabled” spend, on average, double that spent by parents of children without disabilities on living expenses excluding food (Dobson, Middleton, and Beardsworth, 2001, 36) and that the annual cost to parents of ensuring that the minimum essential needs of a child with a disability is met was estimated in both 1998 and 2012 to be closer to around three times higher (Dobson and Middleton, 1998, 1). Thus, as it is the case, too, that double the proportion of parents of children with disabilities are not in paid employment or only in part-time employment compared to parents of children without disabilities (Emerson and Hatton, 2005), find it difficult to sustain paid employment (Dobson, Middleton, and Beardsworth, 2001, 6), and tend to command far fewer resources than those with children without disabilities (Smyth and Robus, 1989), these added costs will often be paid for by the welfare benefits they and their children receive. Similarly, although there are no studies available in the United Kingdom comparing the costs of educating children with special educational needs and disabilities with the costs of educating those without, the government estimates that it costs around seven to nine times more to educate a student in a specialist school than it costs, on average, to educate a child in a mainstream school (Mattingly and McInerney, 2010, 5). It can also be noted that in 2012, in the United Kingdom only 46.3% of working-age persons with disabilities were in employment, compared to 76.4% of working-age nondisabled persons (Statistics South Africa, 2012). Such statistics suggest that persons with disabilities are more likely than persons without disabilities to be dependent on benefits for a large proportion of their income (Wood and Grant, 2010, 34) and thus that the added costs of disability do not necessarily reduce once a child becomes an adult.

However, the picture painted earlier will not apply in all cases of disability, and it may well be the case, as has been noted by Wilkinson, that “for some disabilities . . . lifetime consumption of health and welfare resources is at or below the national average” (Wilkinson, 2010, 101) and thus that the creation of persons who are disabled may be no more likely to burden existing members of society than the creation of nondisabled persons. This point is illustrated well by reference to a study undertaken in the 1990s by Barendregt, Bonneux, and van der Maas which showed that smokers tend, on average, to incur between 7% and 11% less health-care costs over the course of their lifetime than nonsmokers, which means that even before taxes, smokers cost public health-care systems less than nonsmoking citizens (Wilkinson, 2010, 103) because “smoking tends to cause few problems during a person’s productive years and then kills them before social security and pensions payments are made” (Persaud, 1995, 284). We can note, therefore, that persons with certain late-onset genetic disorders, disabilities, and propensities toward certain illnesses, in virtue of their conditions causing them few problems during their childhood and productive years, may end up contributing far more and costing far less, in terms of social and health-care resources, than the average citizen. In such cases, arguments for a duty to select against disability based on the costs associated with accommodating disability will not apply.^14^

The second of our questions is more theoretical. For, *when* it can be shown in a particular case that selection for disability is liable to involve added costs that are not justified by appeals to the claims of justice of the parents themselves or made irrelevant by the production of other benefits, it must be shown, too, that these extra costs are not themselves the result of injustice. How we respond to this question is determined by the extent we hold disability itself to be maladaptive—inherently limiting “the range of opportunity open to the individual in which he may construct his plan of life or conception of the good” (Daniels, 1985, 27)—socially constructed—“a result of a failure to account for everyone when designing physical, economic and social institutions” (Asch, 2003, 319)—or a mixture of the two. For, should we subscribe to the former view, it is understandable that we might hold those who choose to bring into the world lives liable to impose a burden on other members of society morally and financially responsible for their choices.

Whether we should subscribe to the latter or a mixed view, however, that we should condemn those who make such a decision when they might have chosen differently becomes less apparent. For, on such views, disability and its costs are, to some greater or lesser extent, nonnormative and external to the individual, fixed by one’s status as member of a minority group. On such views, the costs associated with accommodating disability are akin to the costs of ensuring the equal participation of black persons and women in a historically racist/sexist society. They are the result of decades, if not centuries, of thoughtless and discriminatory choices on the part of policy makers that are expensive to retrofit. While the costs are real, they are at least partly of our own making and should arguably be borne not only by those who choose to reproduce but also by those who benefit from and are responsible for the existence of such social structures.

After all, it would seem foolish to design our social structures in such a way which means that the basic needs of the minority are cheaper to meet than the basic needs of the majority, and unrealistic, in a society with finite resources, to expect that we should design our social institutions in such a way which means that all can access them, if to do so would be prohibitively expensive. For, if we assume that a basic requirement of a just society is that all reach certain level of some particular currency of justice (welfare, resources, capabilities, etc.) and note, too, that we have limited means of achieving this distribution, it seems that in order to use our resources wisely, we must ensure a just distribution that is also maximally efficient. In many cases, of course, access and opportunity for persons with disabilities and persons without are compatible with one another, and social institutions can be designed that serve both groups equally well. However, in others it makes more economic sense to design such structures in ways that fit the needs of the majority and retain sufficient resources to make adjustments for the disabled so as to ensure full participation by those whose needs differ from the norm and, where this is not possible, to provide such individuals with compensation, which, of course, both explains and justifies the added expense of disability in cases where the added costs of disability are the result not of individual limitation but of the structure of social institutions.

## VI. CONCLUSION

Should prospective parents select against disability in their offspring in situations where they have a choice? In recent years, this question has been addressed in great depth, and from a number of different angles, by scholars concerned with questions of reproductive ethics. Some have asked whether we might ground an obligation to select against disability in the interests of the children we may create, others in notions of what it means to be a good or virtuous parent, and others still in the impersonal claim that those who choose not to select against disability choose to make the world a poorer place than it might have been, had they chosen differently. Within this paper, however, rather than focusing on the above-mentioned arguments, I chose to explore and examine a relatively neglected family of arguments that focus on the real or perceived person-affecting harms that a failure to select against disability may impose on “others” than the child created.

This was done for a number of reasons. First, I aimed to fill a gap in the literature by developing and exploring arguments often mentioned in passing but rarely fully unpacked or examined. Second, I wished to show that despite failure in certain contexts due to the conclusions of the nonidentity problem, appeals to person-affecting harms need not be abandoned in favor of impersonal and duty- or virtue-based arguments when discussing questions of the rights and wrongs of reproduction. Third and finally, I also wished to move away from the belief that reproductive activities are in some sense sacred and that our reproductive choices should thus be immune from the criticisms often faced by other activities based on the externalities they produce. With this in mind, within this paper three possible arguments for a limited moral obligation to select against disability based on person-affecting harms to those other than the fetus were identified and subsequently explored.

The first argument was rather narrow in nature, focusing on the potential harms that prospective parents may impose on themselves as a result of a decision not to select against disability. Appeals to such harms as a basis for an obligation to select against disability, however, were found wanting for three reasons. First, it was noted that procreative desires are not necessarily based in considerations of self-interest, and thus whether or not a decision to procreate is liable to be harmful will not necessarily be relevant in cases where decisions to procreate are based on considerations of duty or are primarily arational in nature. Second, it was also suggested that basing an obligation on parental harms would require us to subscribe to a remarkably strong account of autonomy because, on conventional (weak) accounts, prospective parents are held to be able to consent to harming themselves and may, in fact, be harmed more greatly by paternalistic interferences. Finally, and most importantly, it was also shown—by appeals to research comparing indicators of parental well-being for parents of disabled and non-disabled children—that it is far from clear that decisions not to select against disability are actually liable, on balance, to be any more harmful than decisions to do so.

The second and third arguments, which focused on the “burdens” that decisions not to select against disability may impose on others such as siblings, other dependents, taxpayers, and those whose entitlements to public assistance may be diminished by such a choice, were found to be more promising. For, while parents may accept the harms they might impose on themselves as a result of their reproductive choices, and it is generally held that resources (financial and otherwise) should often be distributed unequally between persons with and persons without a disability, when it comes to assessing the rightness or wrongness of a particular procreative choice, this is irrelevant. Instead, in such cases we must compare “(1) the effects of that choice on each person, against (2) the effects of *each alternative choice*, including those that *exclude* bringing [the child with a disability] into existence” (Roberts, 2009, 29).

Regarding the impact that a decision to bring to birth a child with a disability may have on a particular reproducer’s existing children and other dependents, it was noted that the added expense and other parental resources that a disabled child may require could serve to diminish the welfare of other dependents. Similarly, regarding the interests of other members of the society, it was noted that a decision not to select against disability, at least in societies with socialized medical and welfare systems, is more likely to impose a significant burden of assistance and financial support than a choice to bring to birth a nondisabled child and that this may be problematic on desert-sensitive accounts of moral obligation.

However, while this is so, it was also shown in regard to both arguments that whether or not consideration of such harms will be decisive is actually *highly* contingent on the circumstances surrounding such choices. These include, but are not limited to, considerations of the nature and severity of the disability in question, whether or not its severity can be determined with any accuracy prior to birth, the kinds and extent of the burdens that selection against disability may impose on the parents themselves, the limits of partial parental obligations to their dependents, and the extent to which the added costs associated with a particular disability can be shown to be the result not of individual impairment or limitation but of injustice.

With the above in mind, it is suggested that although there cannot, on the part of prospective parents, exist a general moral obligation to select against disability on the basis of harm to others, there will be scenarios in which appeals to such harms can provide significant, although not decisive, reason to select against disability in their offspring. Appeals to other-regarding harms seem then to result in a rather complex answer to this question when they are given the space and thought required for a full exploration. This, therefore, may explain the reluctance within the philosophical community to employ arguments resting on this basis. However, despite this, consideration of the balance of harms and benefits produced by reproductive choices does offer valuable insight into, and provides sensible and sensitive moral prescriptions for, the diffcult decisions that must be made in the reproductive arena.

## Footnotes

1. Those who make such arguments have suggested variously, although this is by no means an exhaustive list, that selective reproduction will be morally impermissible in all/the vast majority of situations in virtue of the special moral status of the human embryo/fetus (see, e.g., Singer, 2011, 125, for an exploration of the classic pro-life argument and Marquis, 1989, for an example of the potentiality argument for the full moral status of the human embryo), that selective reproduction is offensive to those whose traits are selected against—sending out the message that they are of less value than those with different traits (for an example of this argument, see Asch, 2003), that selection treats our prospective children as a means to an end and is thus incompatible with the virtues one often associates with parenthood (see, for example, Parens and Asch, 1999. and Vehmas, 2001), and that the availability of such technologies may serve to degrade both the parent–child relationship and common conceptions of justice and the relationships that obtain between different members of society (see e.g., Sandel, 2007, 49–50).

2 The International Classification of Functioning, Disability and Health (ICF) defines disability as “an umbrella term covering impairments, activity limitations and participation restrictions . . . a complex phenomenon, reflecting the interaction between features of a person’s body [such as impairments and health conditions like cerebral palsy, Down syndrome and depression] and features of the society in which he or she lives [such as negative attitudes, inaccessible transportation and public buildings, and limited social supports]” (World Health Organization, 2016a. For more information, see World Health Organization, 2016b).

3 According to section 6 (1) of the 2010 Equality Act “A person (P) has a disability if- (a) P has a physical or mental impairment, and (b) the impairment has a substantial and long-term adverse effect on P’s ability to carry out normal day-to-day activities” (Parliament of the United Kingdom, 2010).

4 Of course, as is the case with any normative claim, its prescriptions will tend to adhere to the following general rule of thumb: the more relevant and weighty competing moral claims are to be taken into account, the less likely they are to be, and the less competing and weighty moral claims are to be taken into account, the more likely they are to be. A commitment to the selection against disability view—*if the reasons behind that commitment are defensible*—will lead to an obligation to select against disability in a specific situation. Thus, to use a rather crude example, it can be assumed when applied to different forms of selection that moral reasons in favor of selection against disability will likely hold more weight in a scenario where a couple or single reproducer is already undergoing in vitro fertilization, has no moral objection to the discarding of embryos and is, however implausibly, offered preimplantation genetic diagnosis in order to select against disability for free than in one where fetal abnormality is discovered late in pregnancy where the only option to select against disability is late-term abortion.

5 I note “if at all” in virtue of the fact that the appropriateness of appeals to a harm threshold in cases of disability avoidance has recently been called into question by Anna Smajdor who suggests that such appeals are inappropriate on comparative accounts of harm such as that of Feinberg. For an insight into this lively debate, see Smajdor (2014) and Williams and Harris (2014).

6 Although it is acknowledged here that some may view that the fetus should be counted among persons already in existence and thus included in calculations of harm and benefit in these scenarios, this paper holds, in line with psychological accounts of personal identity over time—and sentience—and personhood-based criteria for moral status, that the fetus/embryo is not an entity of a kind whose interests may be affected by acts of selection. This is so because, while the interests of a fetus in the second or third trimester may be affected by acts causing it harm, the fetus/embryo, in lacking both the capacities required for an interest in avoiding death and for psychological connectedness to the person its organism *could* become, is not an appropriate subject for inclusion in a harm–benefit analysis.

7. A fantastic survey of arguments supporting disability avoidance can be found in Anstey (2008). An equally useful survey of the selection for disability debate can be found in Fahmy (2011).

8. I note that this is largely the case, as opposed to wholly so, in virtue of the fact that a small number of philosophers have attempted to justify a version of the selection against disability view by consideration of such harms. The most notable of such attempts can be found in Douglas and Devolder (2013); Elster (2011); and Roberts (2009).

9. Examples include the discussions surrounding zero-population growth in the 1960s and 1970s and the debates regarding questions of parental justice that raged during the 1990s and early 2000s.

10. See, for example, Casal and Williams (1995), Folbre (1994), George (1987), Olsaretti (2013), Rakowski (1991), and (2002); and Vallentyne (2002).

11. Examples include those who are born into situations of poverty, lack educational opportunities, are discriminated against based on characteristics beyond their control, or lack marketable talents, and so on.

12. Examples include those who are born into rich nations and families, possess numerous valued natural talents and capacities and have been given the opportunities to develop them, or are well educated, and so on.

13. The terms cannot or will not are used here to acknowledge that arguments attempting to ground an obligation to select against disability in order to avoid the imposition of significant and unnecessary costs on others must necessarily be limited in their application only to those who are unable or unwilling to absorb the extra costs associated with a choice not to do so.

14. Indeed, even in cases where evidence points to the conclusion that bringing to birth a child with a particular disability is liable to impose a significant burden of support on others, it should be acknowledged, as was pointed out by an anonymous reviewer in an earlier incarnation of this paper, that in many cases the costs associated with the provision of items such as mobility equipment will be misleading and cannot be accurately assessed by appeals to current expenditure. For, they are often unnecessarily inflated by legal requirements that set standards for the safety and functionality to the needs of the most severely disabled rather than to the needs of the individual, which thus leads to a situation where individuals are provided with equipment that is not only more expensive than it might be but may in fact make the equipment less functional than it could be.
